# PERCEIVED BARRIERS TO AND FACILITATORS OF BEHAVIOURAL CHANGE TOWARDS A MORE ACTIVE LIFESTYLE IN PEOPLE WITH NEUROMUSCULAR DISEASES: A QUALITATIVE STUDY

**DOI:** 10.2340/jrm.v57.42577

**Published:** 2025-03-19

**Authors:** Eric L. VOORN, Sander OORSCHOT, Roos RITMEESTER, Lois DE ZEEUW, Sandra DE MORÉE, Fieke S. KOOPMAN, Annerieke C. VAN GROENESTIJN, Judith G.M. JELSMA

**Affiliations:** 1 Department of Rehabilitation Medicine, Amsterdam UMC location University of Amsterdam, Amsterdam, The Netherlands; 2 Rehabilitation & Development, Amsterdam Movement Sciences, Amsterdam, The Netherlands; 3 Department of Public and Occupational Health, Amsterdam UMC, Vrije Universiteit Amsterdam, Amsterdam, The Netherlands; 4 Health Behaviors & Chronic Diseases, Amsterdam Public Health Research Institute, Amsterdam, The Netherlands; 5 Department of Medical Psychology, Amsterdam UMC location University of Amsterdam, Amsterdam, The Netherlands

**Keywords:** neuromuscular disorders, rehabilitation, qualitative research, healthy lifestyle, behavioural change

## Abstract

**Objective:**

To explore perceived barriers to and facilitators of behaviour change towards a more active lifestyle in people with neuromuscular diseases.

**Design:**

A qualitative study.

**Subjects:**

Nineteen subjects (63% females, age range 28–73 years), representing 4 different neuromuscular diseases.

**Methods:**

Data from a randomized controlled trial were used. Subjects followed a physical activity programme including coaching sessions using motivational interviewing techniques. All sessions were audio-recorded, and thematic analyses were conducted on a random selection of 29 audio recordings, using the International Classification of Functioning, Disability and Health as a framework.

**Results:**

Barriers and facilitators were identified in the following domains: body functions and structures (i.e., neuromusculoskeletal, sensory and mental functions), activities and participation (i.e., undertaking multiple tasks and complex interpersonal interactions), environmental factors (i.e., products and technology for personal use in daily living, design/construction of buildings for public and private use, financial assets, climate, natural events, support and relationships) and personal factors (i.e., satisfaction with life, attitude toward health and disease, attitude toward intervention, exercise habits and methodical skills).

**Conclusion:**

Identified barriers and facilitators could guide healthcare professionals to facilitate the discussion of physical activity behaviour and to address them in a personalized way during neuromuscular rehabilitation treatment.

## LAY ABSTRACT

This study investigated the challenges and factors that help people with neuromuscular diseases to adopt an active lifestyle. Nineteen participants (aged 28–73) with different neuromuscular diseases took part in a physical activity programme that included coaching sessions to explore their own motivation for change. By analysing audio recordings of coaching sessions, we identified key challenges and factors that help people to adopt a more active lifestyle in 4 areas: body functions (such as muscle function, pain, and fatigue), activities (such as undertaking multiple tasks and social interactions), environmental factors (such as accessibility of sports facilities, finances, and social support) and personal factors (such as life satisfaction and attitude to a healthy lifestyle). Understanding these challenges and the factors that help can support healthcare providers to provide more personalized advice and promote physical activity as part of the treatment of people with neuromuscular diseases, together with community services and policy-makers.

With growing evidence of the beneficial effects of physical activity and exercise on various neuromuscular diseases (NMD) (1–3), promoting physical activity has become an important goal of neuromuscular rehabilitation treatment (4), in order to improve well-being and to reduce the risk of non-communicable diseases such as heart disease, stroke, and diabetes. However, people with NMD find it difficult to incorporate regular physical activity into their lifestyle.

Several studies have shown that people living with NMD do not meet international recommendations for physical activity, and that they are less physically active and have a higher risk of developing metabolic syndrome than apparently healthy people without a diagnosis of a neuromuscular disease (5–13). In addition, difficulty with exercise was one of the top 3 “very significant” problems reported by people living with NMD (14), and integrating an exercise programme into daily life was identified as a top priority in their research agenda (15). In line with this, the few exercise studies with long-term follow-up of continuation of aerobic exercise programmes found that 30% to 54% of the participants had stopped exercising up to 3 months after the programme (16, 17).

Unfortunately, little is known about why people living with NMD find it difficult to incorporate regular physical activity into their lifestyle. The few studies that have been conducted on this topic have mainly focused on identifying barriers to physical activity using a quantitative approach (6, 13, 18). Fatigue, physical impairments, and costs have been identified as the main barriers to physical activity in NMD. A better qualitative understanding of potential factors that facilitate or limit physical activity could provide important information needed to develop successful interventions to promote physical activity in NMD (19, 20).

Therefore, the aim of this study was to use a qualitative approach to further explore and describe the perceived barriers to and facilitators of behaviour change towards a more active lifestyle in people living with slowly progressive NMD.

## METHODS

### Design

We used a qualitative research approach, with the data that were collected as part of an ongoing randomized controlled trial on the efficacy of a combined aerobic exercise and coaching programme to improve physical fitness in NMD (Netherlands Trial Register, NL7344) (21). We used data from participants allocated to an aerobic exercise and coaching programme and for whom at least 1 of the sessions of the coaching programme was recorded. The Medical Ethics Committee of Amsterdam UMC, location Academic Medical Center (AMC), approved the study protocol (NL62104.018.17), and written informed consent was obtained from all participants. This study was reported in accordance with the consolidated criteria for reporting qualitative research (22).

### Participants

Participants were recruited from Dutch hospitals and rehabilitation centres throughout the country. Inclusion criteria were: diagnosed with an NMD for which no causative treatment was available; motivated to improve reduced physical fitness; and aged ≥18 years. We recruited various NMD patients, with a focus on post-polio syndrome (PPS), Charcot-Marie-Tooth disease (CMT), and muscular dystrophies and myopathies. Exclusion criteria were contraindications for physical activity according to the American College of Sports Medicine (ACSM) guidelines; unable to follow verbal or written Dutch instructions; engagement in a training programme for a period longer than 4 weeks during the past 6 months.

### Procedures

Data on demographic characteristics (e.g. age, sex, BMI, ethnicity), disease characteristics (e.g., diagnosis, muscle strength by manual muscle testing according to the Medical Research Council scale), and daily steps were collected as part of the randomized controlled trial’s baseline assessment. Daily steps were measured for 7 consecutive days using an accelerometer (ActiGraph GT3X+ accelerometer, Health One Technology, Fort Walton Beach, FL, USA). Average daily steps were calculated if at least 4 days of 8-h data were available.

### Coaching programme

A detailed description of the coaching programme can be found in the published study protocol (21). In short, the coaching programme focused on education concerning an active lifestyle, goal setting, objective monitoring of and feedback on daily activity, and personal coaching, using motivational interviewing techniques to explore the person’s own motivation for change (23). The 26-week coaching programme was guided by trained healthcare professionals, consisting of 8 individual face-to-face sessions and 3 telephone sessions (45 to 60 min), whereby each week a different topic was discussed in more detail (Table SI). Alongside the programme participants received a Fitbit (Fitbit Flex, Fitbit Inc., San Francisco, CA, USA) to provide feedback on the physical activity level.

The coaching programme was guided by occupational therapists or movement therapists, who, prior to the start of the trial, followed a basic course in motivational interviewing, and a 1-day refresher course. To further optimize coaching quality, all therapists received feedback from an experienced motivational interviewing assessor, based on an audio recording of a practice coaching session. Furthermore, therapists were provided with a manual containing an overview of the programme, the content of individual weekly coaching sessions, and instructions on the use of the Fitbit.

### Data collection

All sessions of the coaching programme were digitally recorded (Olympus WS-852, Olympus, Hoofddorp, The Netherlands) and data were stored on a computer for offline analyses. We used a random selection of the available audio recordings. One or 2 recordings per participant were transcribed verbatim and pseudonymized (i.e., confidentially).

### Data analysis

A software package for qualitative data analysis (MAXQDA 2022, VERBI GmbH, Berlin, Germany) was used for thematic data analysis following the 5-step method as described by Castleberry and Nolen (24). In the first step the transcripts were transcribed and read independently several times by 2 researchers (RR and LdZ) for familiarization (i.e., compiling). In the second step (i.e., disassembling) the data were open coded systematically, without a predefined scheme, in 6 transcripts. These codes were clustered in overarching codes during a consensus meeting (RR, LDZ, and JJ) and described in a codebook. This codebook was used to (re)code all transcripts. In the third step (i.e., reassembling) all codes were placed in context with each other to create themes and subthemes with detailed descriptions (RR, LDZ). In the fourth step (i.e., interpreting) all (sub)themes were interpreted in relation to each other and in the light of the available literature and according to the International Classification of Functioning, Disability and Health (ICF) (25) and the systematic classification of personal factors by Grotkamp et al. in 2012 (26), as the ICF does not specify personal factors (JJ, EV, FK, AvG). According to Grotkamp et al., “personal factors” refer to “*features of the individuals that are not part of a health condition or health state*”, as such categorization was done under “functioning and disability” if it was pathology related – otherwise it was considered a “personal factor”. In the final step (i.e., concluding) conclusions were formulated in response to the purpose of the study (JJ and EV). All authors involved in data analysis are experienced in qualitative research.

Descriptive data regarding participant characteristics were analysed using SPSS (version 28.0, IBM Corp, Armonk, NY, USA) and expressed as mean and standard deviation (SD) in case of normally distributed data or as median and range if data were not normally distributed. We visually checked normality based on the histograms of the different variables.

## RESULTS

Between October 2018 and April 2022, 91 participants were randomized, of whom 44 were allocated to the intervention group. We analysed 29 audio recordings of 19 unique participants. All included participants were ambulatory (median daily steps, 4,620; range 1,222–11,672), affected in their lower limbs, and 10 participants used assistive devices and/or orthoses during walking (Table I). The median age of participants was 65 years (range 28–73) and 63% of the participants were female. Eight participants were employed and 6 received their old-age pension. All but 3 participants were Caucasian, and 11 participants were married and/or living together.

**Table I:** Demographic and disease characteristics, and sessions analysed

ID	Age (years)	Sex	Ethnic background	Married/living together	Employee/self-employed	BMI	Diagnosis	MMT sum LE*	Assistive device/orthosis	Daily steps	Sessions analysed
2	50	Male	Caucasian	Yes	Yes	24	CMT2	60.0	Cane, AFO	4735	3 & 5
4	62	Male	Caucasian	Yes	Yes	26	CMT1	67.0		4958	3
6	58	Female	Non-Caucasian	Yes	No	37	CMT1	49.5	Crutch, AFO	1724	2
8	52	Female	Caucasian	No	No	20	CMT1	74.5	Cane	4172	2
9	59	Female	Caucasian	No	No	26	CMT1	71.0	Cane, wheeled walker, AFO	4620	6 & 11
10	65	Male	Caucasian	Yes	No	30	PPS	67.5	Cane, wheeled walker	4964	4 & 9
11	54	Female	Caucasian	Yes	Yes	21	CMT1	74.0		7137	9
15	71	Female	Caucasian	No	No	30	PPS	54.0	Crutch, wheeled walker, KAFO	2820	1 & 3
16	65	Male	Caucasian	Yes	No	23	CMT2	68.5	AFO	6395	9 & 10
18	28	Female	Caucasian	No	Yes	19	CMTuk	70.5		3985	2 & 3
19	65	Female	Caucasian	Yes	Yes	21	PPS	75.5		6621	5
21	68	Male	Non-Caucasian	No	No	22	PPS	56.0	Cane, wheeled walker, AFO	2296	4 & 8
26	34	Female	Non-Caucasian	No	Yes	18	CMT1	77.0		1995	3 & 6
27	41	Female	Caucasian	No	Yes	21	CMT1	76.0		5597	2 & 3
29	73	Male	Caucasian	Yes	No	30	IBM	73.5	Wheeled walker, AFO	1222	1
30	68	Female	Caucasian	Yes	No	28	DM1	75.0			1
32	66	Female	Caucasian	No	No	22	PPS	64.0	KAFO	2337	1
36	65	Female	Caucasian	No	No	26	CMT1	60.0			1 & 2
37	65	Male	Caucasian	Yes	Yes	21	CMT1	76.0		11672	3

AFO: ankle foot orthosis; BMI: body mass index; CMT1: Charcot-Marie-Tooth disease type 1; CMT2: Charcot-Marie-Tooth disease type 2; CMTuk: Charcot-Marie-Tooth disease subtype unknown; DM1: myotonic dystrophy type 1; IBM: inclusion body myositis; KAFO: knee ankle foot orthosis; MMT: manual muscle testing; LE: lower extremities; PPS: post-polio syndrome.

Missing data: daily steps in 2 participants.

* Defined as the sum of scores from 8 lower extremity muscle groups on both sides. Each muscle group had a score between 0 and 5, so that the sum score ranged from 0 to 80.

### Barriers and facilitators

**Fig. 1:**
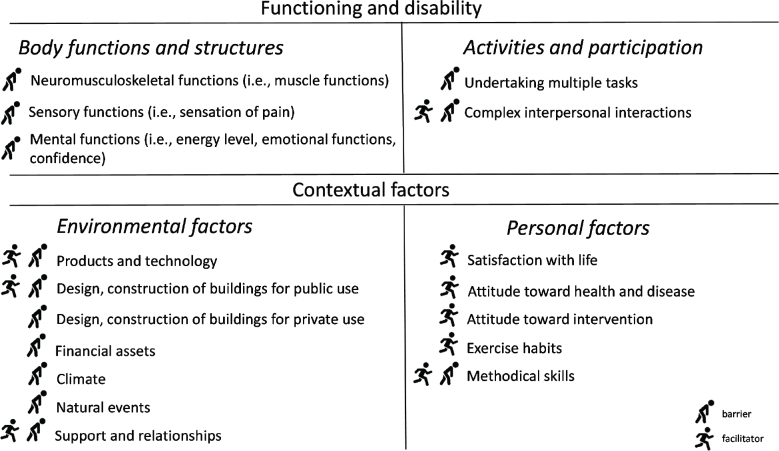
Clustering of barriers and facilitators to physical activity according to the International Classification of Functioning, Disability and Health, for people living with neuromuscular diseases.

Results are presented according to the major ICF components. The major components of “functioning and disability” are “body functions and structures” and “activities and participation”. The major components of “contextual factors” are “environmental factors” and “personal factors”. Fig. 1 contains an overview of the identified barriers and facilitators.

### Functioning and disability

*Body functions and structures.* Barriers to physical activity on the level of “body functions and structures” were related to neuromusculoskeletal and movement-related functions (i.e., muscle functions), sensory functions (i.e., sensation of pain), and mental functions (i.e., energy level, emotional functions, and confidence).

*Muscle functions, unspecified (b749)*. Participants mentioned explicitly or implicitly that everything they did required a constant awareness of their muscle disease: “It is about finding a balance between staying physically active, adapting to the load that the muscles can take, and not being too easily tempted to avoid it or do it too lightly [with less weight]” (ID#16), and that their muscles were not responding as they used to: “In the past, once I started walking, I would naturally warm up…. But now you can just feel your legs getting cold, which makes it harder to move. But yeah, that is just related to polio and muscle diseases, of course” (ID#10).

*Sensation of pain (b280).* The sensation of pain increased the difficulty of sustaining certain physical activities, such as walking with a stick: “My hands hurt. Then I think I generally hold it [the walking stick] too tightly. So that affects walking”(ID#10).

*Energy level (b1300).* Participants described that the constant feeling of tiredness (i.e., low energy level) as a consequence of their disease and physical disability increased the threshold for being (more) active: “I am always tired … I know that you get physically tired if you do little, but it also has to do with the limitations I have” (ID#6).

*Emotional functions (b152).* Many participants indicated that they were afraid to trip or fall: “At the moment I am so terrified to fall” (ID#29). An actual experience of a fall amplified this feeling. Some participants tried to overcome these fears, while other participants avoided these situations or activities: “Walking … I love to be in nature, but it doesn’t give a satisfying feeling, because I am afraid I will trip or fall. Walking is not relaxing anymore” (ID#27).

*Confidence (b1266).* Participants stated that they felt insecure when compared with other “healthy” people at work, during (team)sports or just while walking with friends: “As a child, I was required to do all of that, but I always felt like the clumsy one, the inferior one. And that caused me more stress than enjoyment because I am limited in that regard” (ID#27). The insecurity of not making headway due to their tiredness, pain, and clumsiness made them avoid certain (team)sports and exercise less.

### Activities and participation

Barriers to physical activity on the level of “activities and participation” included undertaking multiple tasks. Complex interpersonal interactions may serve as a facilitator as well as a barrier.

*Undertaking multiple tasks (d220).* Participants mentioned that it was difficult to plan their lives effectively and harmonize different aspects of their lives (e.g., work, family, healthcare, exercise) and multiple tasks: “What you said last time about my work, that has made me think. It is true, those are really exhausting days. And then I do not have the energy to be more physically active” (ID#27).

*Complex interpersonal interactions (d720).* Many participants mentioned that they tried to keep up with others when participating in sports and (social) activities. These interpersonal interactions were considered both a facilitator (being stimulated) and a barrier (exceeding limits): “When someone on a treadmill next to me walks on, for example, a pace of eight, I want to do that too” (ID#32).

### Contextual factors

*Environmental factors.* Barriers to physical activity on the level of “environmental factors” included products and technology for personal use in daily living; design, construction of buildings for public and private use; financial assets; climate; support and relationships; and natural events. Products and technology, construction of buildings for public use, and support and relationships were also seen as facilitators.

*Products and technology for personal use in daily living (e115).* Several participants experienced that their special orthopaedic shoes that should assist in walking also limited them during certain motions or exercises. Moreover, the participants felt as if the risk of tripping and falling increased in certain situations like walking up/down stairs or walking on uneven ground: “with the special shoes I have, especially walking down the stairs is not so easy” (ID#37). On the other hand, participants mentioned they used mobility devices that helped with being active, such as walking poles or frames, and electric bicycles: “And further I have my scooter, but also bring along walking poles and that sort of thing…. I will be more aware that if I can, I should walk a little bit” (ID#10). Also tools such as an activity tracker, which was provided during the I’M FINE programme, was found by many participants to be a facilitator to being active. As the device gave insight into their daily activity, most felt motivated to aim for a certain step goal: “And on some days I see oh, I don’t have that many steps. Then I can think oh no, today I have not been active enough…. But OK, then I still go out for a walk in the evening [to reach my step goal]. So, it is motivating” (ID#18).

*Design, construction, and building products and technology of buildings for public use (e155).* Participants made use of adapted facilities, for example in a swimming pool: “With very low, very small differences in steps, you could walk in the swimming pool. So, I could now enter the swimming pool” (ID#29). On the other hand, participants also experienced a lack of adapted facilities for public use that served as barrier: “At the rehabilitation centre they have some larger changing cubicles and that’s just more comfortable with getting dressed and undressed than in the public swimming pool” (ID#15).

*Design, construction, and building products and technology of buildings for private use (e155).* A lack of space at home prevented some of the participants from being active: “I live in a home where I don’t have a lot of space. I always bump into something when I try to exercise. I have a tiny house” (ID#36).

*Financial assets (e1650).* A few participants indicated that the high costs of sport activities outside the I’M FINE programme limited their ability to be physically active: “And I also think it’s expensive that gym, for what I am doing there” (ID#9).

*Climate (e225).* Most participants noted that the seasonal variation and climate changes influenced their ability and motivation to be physically active. Their muscles and joints were hurting more when it was cold: “In the winter, when it is cold, everything cramps. I find that very difficult” (ID#36). Moreover, when it was cold, rainy or slippery the participants were less inclined to go outside for a walk or bike ride: “Yes, the point is, when the weather gets bad, and in the winter, outside activities will also be less” (ID#21), with a higher risk of tripping and falling in cold weather: “When everything is snowy and slippery, then I won’t go outside as much…. It is just not safe for me” (ID#36).

*Natural events (e230).* A few participants noted that the measures due to the COVID-19 pandemic, such as the multiple lockdowns, restricted their abilities to be more active: “Corona made it impossible to go to the swimming pool, because everything was closed” (ID#32).

*Support and relationships, immediate family (e310).* Participants experienced support from their direct family to stimulate physical activity: “I have my daughters, and my husband. Look, sometimes they do push me [to be physically active]” (ID#6). Too much focus on stimulating physical activity may also work in the opposite direction: “but sometimes, um, they push me too much” (ID#6).

*Support and relationships, health professional (e355).* Some participants were frustrated with the lack of proper guidance:. “Not much is known about the muscle disease and possible remedies. Often it is not understood what people have to endure, even after an explanation. Everyone has different complaints and what I have experienced until now is that people just can’t help me” (ID#8). However, other participants had positive experiences with healthcare professionals and were able to participate in new activities: “With help from a professional I learned to use my legs properly. Before that, I always swam with my arms and my legs just dangled behind me”(ID#15).

*Support and relationships, unspecified (e399).* Exercising together with someone else or in a group setting was found to be more enjoyable and supportive: “I definitely like doing it. I like doing it together” (ID#37). However, some participants mentioned that they lacked social support to keep their motivation to stay active and not enter a downward spiral of negative emotions and consequently be less physically active: “I had nobody that could listen to me and see that it was not going well” (ID#36). Other participants preferred to exercise alone, so they were able to exercise at their own pace and competence level.

### Personal factors

Facilitators of physical activity on the level of “personal factors” included satisfaction with life; attitude toward health and disease; attitude toward intervention and health-related assistance; and exercise habits. Methodical skills were considered both a facilitator and a barrier.

*Satisfaction with life (i413).* Many participants said that it was very difficult and frustrating to acknowledge how the disease had affected their lives. Accepting these aspects provided some relief and made the situation more manageable. This was reflected, for example, in the changed use of the Fitbit activity tracker: “And now I can more easily accept how it’s going. Because then I often think oh but that was the reason [too many steps] why it wasn’t going as well or something like that” (ID#10).

*Attitude towards health and disease (i416).* Many participants had a desire to improve their health, fitness and overall (active) lifestyle: “Well I also think it will improve my fitness. Exercise is good, so in that sense it’s good” (ID#16). Besides knowing the health benefits, the experienced positive changes in endurance level, physical strength, and overall well-being were mentioned as a motivating factor to continue: “and I really couldn’t keep it up longer than 15 minutes, but right now I manage half an hour” (ID#29).

*Attitude towards intervention and health-related assistance (i419).* The prospect of health benefits motivated people to participate in the intervention programme: “So I hope that through this programme [I’M FINE] I can stabilize my physical fitness level. Of course it would be nice if it improves as well” (ID#8).

*Exercise habits (i456).* Exercising was made more fun by listening to music or a podcast, or watching television while on a home trainer: “And I do think a podcast or music is a great invention, especially podcasts – I have only recently started with those. And then you combine something fun with [physical activity]” (ID#18). Furthermore, activities that they (really) enjoyed were easier to maintain: “And especially if it suits me, then I can sustain it yes, there is always something to be found that suits you” (ID#18). Participants also incorporated, as a strategy, physical activity in daily tasks, for example when going to work or the supermarket they would take the bike or walk instead of another means of transport.

*Methodical skills (i433).* Some participants deliberately chose exercise activities that did not put too much strain on the body and that were beneficial for balance and to build strength, such as yoga or swimming: “I like swimming, because in the water you can make movements that I normally cannot make. It feels really good to do that. I used to love doing yoga. But I cannot do that anymore. In the water though, I can do all of it – that is magical” (ID#36). Another strategy commonly used by participants was to create a plan and structure for maintaining an active lifestyle. They planned to have enough rest between activities to replenish energy and be able to participate in multiple activities throughout the day. For instance, one participant said: “When I am finished with work I would rest for a bit and then have dinner and then after that I would go to the gym” (ID#26). Even if they were not always in the mood or perhaps a little too tired to be active, the agenda reminded them to persevere: “That was especially with cycling, that I would really look ahead [and plan] and then I am going to do that. And maybe I should also do that for my spare time, that I make a schedule [for that]” (ID#11). There were also participants for whom difficulties with agenda setting and prioritizing were barriers to physical activity: “I want to be physically active, but I always think, we will do it tomorrow or it will happen [someday]” (ID#6).

## DISCUSSION

This qualitative study explored the perceived barriers to and facilitators of behaviour change towards a more active lifestyle in slowly progressive NMD. Using the ICF as a framework, we identified both barriers and facilitators across all the domains.

On the domain “body functions and structures”, we identified only barriers to physical activity. Despite differences in terminology, as most of the previous studies did not use the ICF as a framework, there is a large similarity in the barriers identified across the studies (6, 13, 18, 20, 27). For example, sensation of pain and energy level, referred to as fatigue in most other studies, are important barriers for people with NMD that have been reported to be more common than in non-disabled people (13). Other barriers we found were emotional functions, more specifically the fear of falling, and confidence. Fear of falling is a common problem for people with PPS and CMT, who often have lower limb muscle weakness (6, 20). People act differently; some avoid certain physical activities, while others try to overcome their fears, in order to remain physically active.

An important barrier in the “activities and participation” domain was the difficulty of fitting physical activity around other aspects of participants’ lives, such as work, family, and healthcare (13, 18, 20). In addition, a factor that we found and that had not previously been reported in NMD was “complex interpersonal interactions”. Participants mentioned this in the context of trying to keep up with others in sports and social activities, which provided motivation, but in other cases led to pushing boundaries. In contrast to other studies, transport was not mentioned as a barrier by participants (6, 13, 18, 27). This may be due to the fact that the Netherlands has good facilities for disabled people to travel longer distances, that our cohort consisted of less affected people compared with other cohorts (27), or that the Netherlands is a relatively small country compared with countries such as Sweden and the United States (18, 20).

The barriers and facilitators related to the “environmental factors” domain were in line with those identified in previous NMD studies (6, 13, 18, 20, 27). These included barriers such as bad weather and the high cost of sports activities. Other aspects, such as support from close relatives and healthcare professionals and accessible environments, or the lack thereof, could act as both facilitators of and barriers to physical activity. Importantly, as our study was conducted during the COVID-19 pandemic, participants were exposed to lockdowns, including the closure of public areas such as physiotherapy practices and gyms. This had a major impact on people’s ability to be physically activity (28), but was very specific to this situation and unlikely to be repeated.

Although not always categorized as such in previous studies, most of the barriers and facilitators in the “personal factors” domain have been reported previously, such as the attitude towards health and disease (e.g., exercise is good), exercise habits (e.g., make physical activity fun), and methodical skills (e.g., planning of physical activity). The notion that our findings are most comparable and consistent with 2 of the previous studies that used the ICF model (20) and the social-ecological model (27) to categorize barriers and facilitators to physical activity indicates that the use of a model could help to provide a more complete picture and to identify the factors that could be addressed by rehabilitation treatment. Despite these similarities, there are also differences in the interpretation of the results. In particular, the categorization of certain barriers and facilitators as “personal factors” implies that we did not consider them to be related to the pathology. For example, while “planning of physical activity’ may well be related to the pathology in a person who has had a stroke and therefore fall into the “body functions and structures” domain, as it may in a person with myotonic dystrophy, this is not necessarily the case in people with other NMD. This may also have implications for rehabilitation treatment.

When our findings are compared with other physical disabilities, such as spinal cord injury, stroke, and cerebral palsy, the barriers and facilitators are generally very similar, with small differences between conditions (29). While attention should be paid to these differences between conditions in rehabilitation treatment, community- and policy-level strategies should focus on the wider population of people with physical disabilities (29, 30).

The current qualitative data provide an in-depth insight into the barriers and facilitators experienced by people with NMD when making behavioural changes, which is unique as intervention sessions are rarely recorded (i.e., black box). As we analysed audio recordings of coaching sessions, our findings were not influenced by the interviewer’s (in our case the coach’s) prior understanding and relationship with the participants. A disadvantage was that it did not allow for follow-up questions, clarification of points, and exploration of emerging themes in the moment. The study cohort came from a randomized controlled trial in which we selected people for a physical activity programme. Although the number of daily steps was lower than in previous cohorts (7, 11, 31, 32), more affected and wheelchair-bound people were under-represented, as we selected participants who could perform an exercise test and who were able to participate in the physical activity programme, which limits generalizability. The classification of “personal factors” in addition to the ICF model was a strength of our study, which may help to provide a more complete picture and to identify modifiable barriers and facilitators.

Barriers to and facilitators of physical activity in NMD are multifactorial, highlighting the need for multidisciplinary neuromuscular rehabilitation treatment. An important first step in changing behaviour towards an active lifestyle is to identify the patient’s personal barriers and facilitators across all ICF domains. Motivational interviewing, which aims to explore the person’s intrinsic motivation for change, can also play an important role in this process. Accordingly, professionals from different paramedical disciplines can contribute to the desired behavioural change by addressing barriers and facilitators in their own area. For example, physiotherapists can focus on regaining confidence by giving advice on how to improve body functions such as endurance and balance so that people are less afraid of falling. Occupational therapists and social workers can provide advice on energy levels, agenda setting, and social interactions, and psychologists can focus on coping strategies such as acceptance of the disease. While healthcare professionals can make an important contribution, they should also recognize that part of the solution lies at the community and policy level: there is a strong need for (inter)national health, transport, architecture, and construction policies to promote physical activity among the general population, but especially among people with NMD and other disabilities (30).

In conclusion, in this qualitative study we found that the barriers to and facilitators of physical activity in NMD are multifactorial. Using the ICF as a framework, there appeared to be barriers and facilitators in all domains of the ICF. This information can be used by healthcare professionals in their daily practice and also to further develop interventions to promote physical activity in NMD, which should focus on rehabilitation, preferably in co-production with community services and policymakers.

## Supplementary Material

PERCEIVED BARRIERS TO AND FACILITATORS OF BEHAVIOURAL CHANGE TOWARDS A MORE ACTIVE LIFESTYLE IN PEOPLE WITH NEUROMUSCULAR DISEASES: A QUALITATIVE STUDY
