# Supplementary material for: PERCEIVED BARRIERS TO AND FACILITATORS OF BEHAVIOURAL CHANGE TOWARDS A MORE ACTIVE LIFESTYLE IN PEOPLE WITH NEUROMUSCULAR DISEASES: A QUALITATIVE STUDY
Source: J Rehabil Med. 2025 Mar 19;57:42577. doi: 10.2340/jrm.v57.42577 (PMC11971938; doi:10.2340/jrm.v57.42577)
Supplement: PERCEIVED BARRIERS TO AND FACILITATORS OF BEHAVIOURAL CHANGE TOWARDS A MORE ACTIVE LIFESTYLE IN PEOPLE WITH NEUROMUSCULAR DISEASES: A QUALITATIVE STUDY [file JRM-57-42577-s1.pdf]

Supplementary material has been published as submitted. It has not been copyedited, or typeset by Journal of Rehabilitation Medicine

Table SI. Coaching programme overview.

Session	Topic	Time (min)
1	Treatment goals and agenda setting coaching programme	60
2	Potential advantages of a healthy lifestyle and insight in the current personal situation	60
3	Explore motivation to change lifestyle	45
4	Setting realistic goals	45
5	Social support, sports counselling center[1]	60
6	Progress 1 (telephone)	15
7	Progress 2 (telephone)	15
8	Progress 3 (telephone)	15
9	Progress and relapse prevention	60
10	How to proceed and completion of coaching programme	45
11	Optional; evaluation and relapse prevention	60

[1] Hoekstra F, Hoekstra T, van der Schans CP, et al. The implementation of a physical activity counseling program in rehabilitation care: findings from the ReSpAct study. *Disability and rehabilitation*, 2021; 43: 1710-1721.
